# Estimating the risk of type-2 diabetes using obese-years in a contemporary population of the Framingham Study

**DOI:** 10.3402/gha.v9.30421

**Published:** 2016-06-30

**Authors:** Asnawi Abdullah, Fauzi Ali Amin, Farida Hanum, Johannes Stoelwinder, Stephanie Tanamas, Rory Wolf, Evelyn Wong, Anna Peeters

**Affiliations:** 1 Faculty of Public Health, University Muhammadiyah Aceh, Banda Aceh, Indonesia; 2 School of Public Health and Preventive Medicine, Monash University, Clayton, Australia; 3 National Institute of Diabetes and Digestive and Kidney Diseases, National Institutes of Health, Bethesda, MD, USA; 4 School of Health & Social Development, Deakin University, Melbourne, Australia

**Keywords:** BMI, obesity, offspring Framingham, obese-years, type-2 diabetes

## Abstract

**Background:**

We have recently demonstrated that an obese-years construct is a better predictor of the risk of diabetes than the severity of body weight alone. However, these risk estimates were derived from a population cohort study initiated in 1948 that might not apply to the current population.

**Objective:**

To validate an obese-years construct in estimating the risk of type-2 diabetes in a more contemporary cohort study.

**Design:**

A total of 5,132 participants of the Framingham Offspring Study, initiated in 1972, were followed up for 45 years. Body mass index (BMI) above 29 kg/m^2^ was multiplied by the number of years lived with obesity at that BMI to define the number of obese-years. Time-dependent Cox regression was used to explore the association.

**Results:**

The risk of type-2 diabetes increased significantly with increase in obese-years. Adjusted hazard ratios increased by 6% (95% CI: 5–7%) per additional 10 points of obese-years. This ratio was observed to be similar in both men and women, but was 4% higher in current smokers than in never/ex-smokers. The Akaike Information Criterion confirmed that the Cox regression model with the obese-years construct was a stronger predictor of the risk of diabetes than a model including either BMI or the duration of obesity alone.

**Conclusions:**

In a contemporary cohort population, it was confirmed that the obese-years construct is strongly associated with an increased risk of type-2 diabetes. This suggests that both severity and the duration of obesity should be considered in future estimations of the burden of disease associated with obesity.

## Introduction

Approximately 1.12 billion individuals globally will become obese in the next decade (1). In some countries, such as the United States, 34.9% (78.6 million) of adults and 17% (38.3 million) of youths are now obese (2). If current secular trends continue, obesity will be a burden on health systems worldwide (3), as it is strongly associated with an increased risk of type-2 diabetes and other chronic diseases (4). A meta-analysis found that people with obesity have a seven-fold higher risk of type-2 diabetes compared to those with normal weight (5). Therefore, not surprisingly, obesity has been blamed for the current epidemic of type-2 diabetes worldwide (6). Today, there are an estimated 415 million people living with diabetes, including 193 million who are undiagnosed, while a further 318 million adults have impaired glucose tolerance, which increases the risk of diabetes. If the current obesity trend continues unabated, diabetes will cause more than 5 million deaths and cost 1,000 billion dollars in healthcare spending (7), threatening global economies in both developed and developing countries (8).

The future burden of diabetes may be underestimated if the adverse effect of obesity is not totally captured. The current analysis of the burden of diseases relies mostly on the severity of obesity, or the duration of obesity only. A combination of both the severity and duration of obesity into a single measurement of obese-years has been ignored. We recently proposed a new approach of calculating the total adverse effect of obesity (9), in which the cumulative risk for a person with mild obesity for a long period of time is considered similar to that for more severe obesity for a shorter period of time. This approach is analogous to smoking-related studies in which the total adverse effects of the combined quantity of cigarettes or packs smoked per day and the duration of smoking, expressed as cigarette-years or pack-years (10–12) has been well recognized.

Recently, two studies have reported that an obese-years construct is a better and more concise estimation the risk of type-2 diabetes (9) and cardiovascular disease (13), compared with a construct based on either the duration or severity of obesity alone. However, those studies have some limitations, as the population used relied on the relatively old prospective cohort of the original Framingham Heart Study (FHS) that began in 1948. In the 1950s, the prevalence of obesity in America was still relatively low (14). Nowadays, the prevalence of obesity is significantly higher. Moreover, the number of years of living with obesity is also increasing as the onset of obesity is occurring earlier, even in childhood. Therefore, the previous estimation of the risk of type-2 diabetes (9) might not apply to current population characteristics.

The primary aim of this study was to calculate the association between the obese-years construct and risk of type-2 diabetes in a more contemporary population cohort study using the Framingham Offspring Study (FOS). In FOS, adult children of the original FHS members and their spouses were followed up from 1971 for over 45 years. During the follow-up, the participants underwent regular physical examinations. The secondary aim was to examine whether a model with the obese-years construct offers a better goodness of fit for estimating the risk of type-2 diabetes than separate models based on either body mass index (BMI) or the duration of obesity alone.

## Methods

### Source of data

We used data from the FOS in this prospective cohort study of the offspring of the original cohort of FHS and their spouses. The original cohort of the FHS began in 1948 and aimed to identify the common risk factors associated with cardiovascular disease (15). In 1971, 3,548 adult children of the original FHS members and 1,576 of their spouses aged of 5–70 years were added into a new study, called the FOS. The main objective of the FOS was to examine the secular changes in the levels of risk factors between the two generations (16). A total of 5,227 participants of the FOS were followed up for approximately 45 years. The latest examination (examination 9) began in April of 2011 and concluded in April of 2014. For this study, data was only available until examination 8.

### Measurement of variables

In the FOS, extensive physical examination, laboratory tests, health behaviors interviews, and medical history and illness conditions were measured regularly, approximately every 3 to 8 years using a standardized protocol. Obesity was defined using the World Health Organization's BMI cut-off point for obesity (17), a BMI of 30 kg/m^2^ or greater. Demographic variables of age, sex, education, and marital status are included in the analysis as covariates. The age of participants was defined as years from date of birth to the date of examination. Education was defined as the number of years of schooling. Marital status was grouped into three categories (single, married, and widowed, divorced or separated) and it was measured at examination 2, 4, 5, 7, and 8. Health behavior variables of smoking status (current smokers and never/ex-smokers) and number of cigarettes smoked per day, number glasses of alcoholic drinks per week (beer, white wine, red wine, or liquor), and number of hours of physical activity were included in the analysis. Current smoker was defined a participant who had smoked regularly in the past year. Alcohol consumption was measured in standards glasses per week. In the FOS, a participant was asked for the number of hours for rest (sleep and sedentary, such as reading watching TV) and physical activity for a typical day (most days of the week) in 24 h. Physical activity included the number of hours of slight activity, such as standing and walking; moderate activity, such as housework (e.g. vacuuming, dusting, doing yard chores, climbing stairs) and light sports (e.g. bowling, golf); and heavy activity, such as heavy household or yard work (e.g. stacking or chopping wood) and exercise and intensive sports (e.g. jogging, swimming). These categories were not mutually exclusive.

### The measurement of obese-years

Obese-years was constructed by combining the severity of obesity and the duration of obesity into a single variable. Obese-years was calculated by the severity of obesity (in ‘obese units’, equivalent to BMI units (kg/m^2^) at each examination by the duration of obesity (the number years lived with obesity). At each examination, the cumulative number of obese-years was computed as the sum of all obese-years ‘exposure’ up to that examination. The severity of obesity was calculated as follows: (1) if BMI <30 kg/m^2^, the severity was zero; and (2) if BMI ≥30 kg/m^2^, the severity was BMI minus 29 kg/m^2^. For example, if BMI was 34 kg/m^2^, the severity was 5 (34) obese units. The number years lived with obesity (obesity duration) was defined from the onset of obesity as the date of the first examination at which the individual was obese and, from that time, the individual was considered to be continuously obese until a non-obese examination, after death, the end of follow-up. A participant could have multiple periods of obesity during the study follow-up.

Table 1 illustrates the calculation of obese-years for a single individual. This participant first had a measurement of obesity at examination 2 and was assigned an obesity duration of zero at this examination. At examination 3, this participant was assumed to have lived with obesity for 4.7 years (the time interval between examinations 2 and 3), with a degree of obesity of 1.5 kg/m^2^. This approach assumes that an individual's BMI is carried forward from a given examination (i.e. examination 2) and does not change until a different BMI value is recorded at a subsequent examination (i.e. examination 3). The number of obese-years at examination 3 was therefore 7.1 obese-years (1.5 BMI unit×4.7 years in the preceding interval). From examinations 3 to 4 (a 3-year interval), the participant was still obese with a degree of obesity of 3.6 kg/m^2^ (BMI 32.6 kg/m^2^). At examination 4, the number of obese-years was 10.8 (3.6 BMI unit×3 years) and the cumulative obese-years at this examination was 17.9 obese-years (7.1 plus 10.8). This method implies that individuals accumulating 40 obese-years, for example, could have reached this quantity either by having been obese with a BMI of 30 kg/m^2^ for approximately 40 years or by having been obese with BMI of 33 kg/m^2^ for approximately 10 years or, indeed, many other combinations.

**Table 1:** Illustration of the calculation of an obese-years metric for a single hypothetical individual

ID	Examination	Date of examination	Interval between examinations, years^a^	Body mass index^b^	Degree of obesity	Duration of obesity, years	Obese-years^a^	Cumulative obese-years
3	1 (baseline)	02-Apr-73	–	28.3	0	–	0	0
3	2	16-Mar-81	8.0	30.5	1.5	0	0	0
3	3	15-Nov-85	4.7	32.6	3.6	4.7	7.1	7.1
3	4	23-Nov-88	3.0	34.4	5.4	3.0	10.8	17.9
3	5	31-Aug-92	3.8	32.7	3.7	3.8	20.5	38.4
3	6	18-Feb-97	4.5	30.3	1.3	4.5	16.7	54.5
3	7	13-Jun-00	3.3	28.1	0	3.3	4.3	59.3
3	8	21-Apr-06	5.9	28.4	0	0	0	59.3

a All intervals refer to the intervals between the prior and current examinations.

b Body mass index: weight (kg)/height (m)^2^.

### Measurement of the outcome and time to event

The main outcome of this analysis was type-2 diabetes. A person was diagnosed with type-2 diabetes if their fasting plasma glucose levels were greater than 126 mg/dL (7 mmol/L), or he or she was treated with insulin or an oral hypoglycemic agent at a given examination. For the purpose of this study, those who had been diagnosed as having type-2 diabetes at baseline (*n=*95) were excluded from analysis. Participants who died, lost to follow-up, or reached the end of the study follow-up (examination 8) without a diabetes diagnosis were censored at the date of death, lost to follow-up, or examination 8.

### Statistics data analysis

A dynamic survival model (18), with time-dependent Cox proportional hazards (19, 20), was deployed. Most variables included in the model were time-varying, except for sex and ethnicity, which did not vary during the study follow-up. To handle the time-varying variables in the analysis, the structure of the dataset was transformed (or reshaped) from wide to long and captured the variation of values of variables during study follow-up, and a time-dependent (extended) Cox regression analysis was performed. In a time-dependent Cox regression, the follow-up time for each participant is divided into different time windows (i.e. the interval of examinations). For each time window, a separate Cox analysis is carried out using the specific value of the time-dependent variable at the beginning of that specific time window. A weighted average of all the time window–specific results is calculated. This weighted average of a series of relatively short-term effects is presented as the result of the analysis as a single hazard ratio (HR). More detail on this approach has been described by Dekker et al. (21).

The obese-years construct was analyzed both as a continuous and categorical variable. The categorical variable was categorized into five groups of obese-years (0, 1–24.9, 25–49.9, 50–74.9, and ≥75). For the continuous variable of obese-years, HRs were presented per additional 10-unit increase of obese-years, where a one-unit increase in obese-years can represent an additional BMI level above ≥30 kg/m^2^ or an additional year lived with obesity at a given BMI level. These categories were used to make the results of this study similar to the categories of previous findings (9, 13), to facilitate comparison. Analyses were performed for total participants but also for categories stratified by gender and smoking status (current and never/ex-smokers,) as previous findings showed some variation in the relationship between obese-years and the risk of type-2 diabetes by gender and by smoking status (9). Two main models were employed to examine the effect of possible confounders on the relationship between the obese-years construct and incident type-2 diabetes. Model 1 was adjusted for the demographic variables of age, sex, marital status, and educational level. Model 2 was adjusted for these demographic variables plus the health behavior variables of smoking, alcohol consumption, physical activity, and family history of type-2 diabetes.

The severity of body weight variable (BMI model), the number of years of exposure to the obesity variable (the duration of the obesity model), and the obese-years variable (the obese-years model) were each grouped into a number of categories. For the BMI model, BMI ≤30 kg/m^2^ (not obese) was used as a reference category. For those with BMI ≥30 kg/m^2^ (obesity), having the duration of obesity (≥1 year) and ≥1 obese-years, 10 categories were defined based on deciles, again, similar to the categories used in the previous study (9) for that sake of comparison. The goodness of fit of the three models was compared using Akaike's information criterion (AIC), computed as −2 (log-likelihood)+2 (number of estimated parameters), with a lower AIC indicating a better fit (22). All statistical analysis was conducted using Stata version 11 (23).

## Results

### Characteristics of the participants

During 152,700 person-years of follow-up, 35% of participants (1,786 out of 5,132 eligible participants) were identified as obese (Table 2). For those who were ever obese during of follow-up, the mean cumulative years lived with obesity was approximately 15 years (range 1–35 years) and the mean cumulative obese-years was 72 (range 2–626 obese-years).

### Incidence rate of type-2 diabetes

**Fig. 1:**
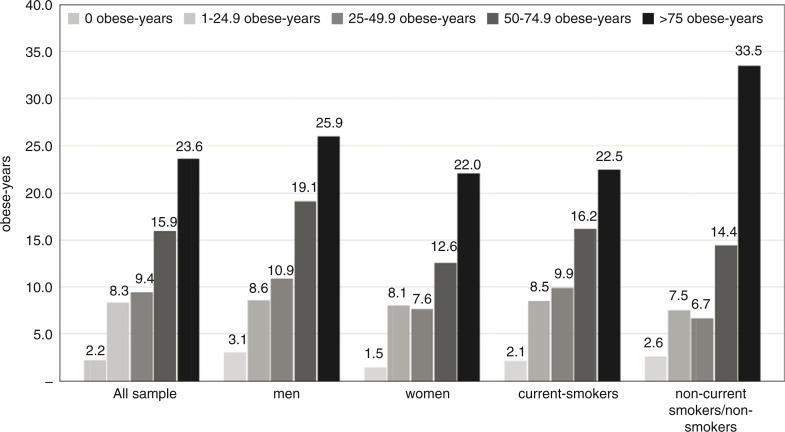
The incidence of type-2 diabetes (events/1,000 person-years), according to cumulative obese-years, overall, and separately stratified by sex and by smoking status.

During the study follow-up, 903 (18%) participants died or 1,127 (22%) were lost to follow-up and 685 (13%) of the participants were diagnosed with type-2 diabetes. A total of 3,544 participants were censored at the end of the study. The incidence rate of type-2 diabetes was 4.5 per 1,000 person-years. The incidence rates of type-2 diabetes increased as the number of obese-years increased and were 2.2, 8.3, 9.4, 15.9, and 23.6 for the obese-years categories of 0, and 1–24.9, 25–49.9, 50–74.9, and 75+ obese-years, respectively (Fig. 1).

**Table 2:** Characteristic of the population included in analysis^a^

Participants characteristics	Number	%	Mean	Range
Eligible sample	5,132			
Participants ever obese during study follow-up^a^	1,786	35		
Age at baseline; years			37	5–85
Sex: men	2,671	52		
Ethnicity: Caucasian	5,121	99		
Marital status				
Single	332	6		
Married	4,417	86		
Widowed, divorced or separated	386	8		
Educational level (year of schooling)			14 years	1–30
Health behavior				
Smoking status at baseline: yes	2,221	43		
Ever smoking during study follow-up	2,480	48		
Physical activity at baseline				
Slight physical activity	2,914	56	5.4 h/day	
Moderate physical activity	2,837	54	3.8 h/day	
Heavy physical activity	1,169	22	1.7 h/day	
Alcohol drinking at baseline; glass/week	1,273	59		
Wine	2,537	49		
Beer	2,429	47		
Cocktail	3,458	67		
Biochemical characteristics				
Serum cholesterol LDL at baseline; mg/100 ml			125	39–403
Serum cholesterol HDL at baseline; mg/100 ml			56	9–132
Fasting Blood glucose at baseline; mg/100 ml			101	60–193
Body weight characteristics				
BMI at baseline; kg/m^2^			25	14–54
Underweight (<18.5 kg/m^2^)	130	3		
Normal weight (18.5–24.9 kg/m^2^)	2,662	52		
Overweight (25–29.9 kg/m^2^)	1,709	33		
Obese (≥30 kg/m^2^)	619	12		
Cumulative obesity duration; years^b^			15	1–35
Cumulative obese-years^b^			72	2–636
Incidence rate of type-2 diabetes per 1,000 person-years	4.5			

a Participants were free from existing diabetes at study baseline.

b For those who were obese during study follow-up.

### Hazard ratios for type-2 diabetes

The adjusted HRs of type-2 diabetes for the categories of 1–24.9, 25–49.9, 50–74.9, and ≥75 obese-years were 2.50 (95% CI: 1.85–3.37), 2.94 (95% CI: 2.04–4.24), 5.09 (95% CI: 3.56–7.26), and 6.05 (95% CI: 4.65–7.89), respectively, compared with zero obese-years (Model 2). A dose–response relationship was observed (*p <* 0.001). When analyzing the continuous variable of obese-years, the HR for the total population was 1.06 (95% CI: 1.05–1.07) per additional 10 obese-years (Table 3). An interaction was observed between obese-years and smoking status (*p <* 0.001), but not with gender.

**Table 3:** The hazard ratio (95% confidence interval) of type-2 diabetes per 10 obese-years or according to obese-years category

	Model 1	Model 2
A. Continuous obese-years^a^		
All sample	1.07 (1.06–1.08)	1.06 (1.05–1.07)
Men	1.07 (1.05–1.09)	1.06 (1.04–1.08)
Women	1.07 (1.06–1.08)	1.06 (1.05–1.07)
Current smokers	1.11 (1.07–1.15)	1.10 (1.06–1.15)
Never/ex-smokers	1.07 (1.06–1.08)	1.06 (1.05–1.07)
B. Categorical obese-years		
All sample		
0	1	1
1–24.9	2.75 (2.06–3.68)	2.50 (1.85–3.37)
25–49.9	3.09 (2.17–4.40)	2.94 (2.04–4.24)
50–74.9	5.45 (3.88–7.65)	5.09 (3.56–7.26)
≥ 75	6.72 (5.20–8.68)	6.05 (4.65–7.89)
*P* value test trend	*p*=0.001	*p*=0.001
Men		
0	1	1
1–24.9	2.26 (1.54–3.31)	2.11 (1.43–3.13)
25–49.9	3.05 (1.96–4.75)	2.90 (1.84–4.57)
50–74.9	5.11 (3.32–7.87)	4.98 (3.19–7.76)
≥ 75	5.02 (3.48–7.24)	4.24 (2.91–6.19)
*P* value test trend	*p*=0.001	*p*=0.001
Women		
0	1	1
1–24.9	3.54 (2.28–5.51)	3.07 (1.90–4.96)
25–49.9	2.99 (1.65–5.43)	2.94 (1.59–5.46)
50–74.9	6.02 (3.48–10.43)	5.27 (2.93–9.46)
≥ 75	8.99 (6.26–12.90)	8.88 (6.14–12.85)
*P* value test trend	*p*=0.001	*p*=0.001
Current smokers		
0	1	1
1–24.9	2.14 (0.89–5.14)	1.89 (0.75–4.80)
25–49.9	3.41 (1.12–10.32)	3.47 (1.15–10.50)
50–74.9	4.49 (1.50–13.43)	4.83 (1.76–13.25)
≥ 75	17.96 (8.80–36.66)	15.50 (6.93–34.67)
*P* value test trend	*p*=0.001	*p*=0.001
Never/ex-smokers		
0	1	1
1–24.9	2.83 (2.08–3.84)	2.59 (1.88–3.55)
25–49.9	3.08 (2.12–4.47)	2.92 (1.99–4.29)
50–74.9	5.44 (3.80–7.79)	5.13 (3.52–7.46)
≥ 75	6.08 (4.63–7.99)	5.54 (4.18–7.35)
*P* value test trend	*p*=0.001	*p*=0.001

Model 1 is adjusted for the age, sex, marital status, and educational level of the entire sample and for the sample of current smokers or never/ex-smokers; for the sample of men or women, it is adjusted for age, marital status, and educational level. Model 2 is adjusted for the age, sex, marital status, educational level, current smoking status, alcohol consumption, physical activity, and family history of diabetes for the entire sample; for the sample of men or women, the model is adjusted for all those variables except the variable of sex; and for current smokers and never/ex-smokers, it is also adjusted for all those variables, except for smoking status.

a Hazard ratios refer to the increase in type-2 diabetes risk associated with each additional 10 obese-years.

### Comparing the different body weight metrics using AIC

Table 4 shows a comparison of the goodness of fit for the models for three different constructs of body weight (obese-years, the duration of obesity and BMI). The models were compared using the AIC, examined separately for each model as an additive effect. In all population groups (total population, men/women, and current smokers and never/ex-smokers), the AIC score was lower in the model with the obese-years construct compared with other models, which suggests that the obese-years metric is the best model for estimating the risk of type-2 diabetes compared with other two models of BMI and the duration of obesity. This superior fit of the obese-years model was particularly observed in women and smokers.

**Table 4:** Comparison the AIC value of BMI, the duration of obesity, and the obese-years metric as predictors of the risk for type-2 diabetes

		Sex		Smoking status	
			
Models	All sample	Men	Women	Current smokers	Never/ex-smokers
BMI	5,675	2,835	2,283	511	4,898
The duration of obesity	5,699	2,852	2,295	525	4,909
Obese-years	5,672	2,842	2,274	510	4,894

Each model analyses the obesity exposure measure categorized into 11 categories; 1=‘not obese’, and 2–11 are deciles of obesity exposure. For the entire sample, the models are adjusted for sex, age, marital status, education, smoking status, alcohol consumption, physical activity, and family history of type-2 diabetes subjects. For the sample of men or women, all the above variables are adjusted for, except the variable of sex. For the sample of current smokers or never/ex-smokers, all the above variables are also adjusted, except the variable of smoking status.

## Discussion

Using a contemporary population cohort study, the FOS, our analysis confirmed our previous findings, using the original cohort of FHS, that an obese-years construct yields a better estimation of the risk of incident type-2 diabetes than a construct based only on either BMI or the duration of obesity. In both the FHS (baseline 1948) and FOS (baseline 1971), a dose-response relationship was clearly observed. For every additional 10 obese-years, the risk of type-2 diabetes increased by approximately 7% (95% CI: 5–9%). This finding has important clinical and public health implications for obesity prevention strategies and future studies of the burden of disease associated with the adverse impact of obesity.

We argue that combining both the severity and the duration of obesity into a single construct of obese-years provided not only a better construct for estimating type-2 diabetes and cardiovascular disease (13), but also a better construct for estimating the risk of all-cause and cause-specific mortality compared to using the current constructs of BMI or obesity duration (24). It is recommended that the usefulness of the obese-years construct should be tested for estimating the health outcomes for other conditions, such as cancer, that have shown a limited association with the severity of obesity (25).

In this study, using a contemporary population cohort, we have observed that there is no difference between men and women in the risk of developing type-2 diabetes. This contrasts with the previous study using the origin cohort of the FHS, which found the risk of type-2 diabetes increased with additional obese-years more strongly for men than for women (9). Although the previous study further showed a relatively similar effect of obese-years for smokers and non-smokers, this analysis found a higher risk of type-2 diabetes associated with the obese-years construct for smokers than for non-smokers. Most smokers tend to be leaner, but weight loss among them appears to signal a greater amount of smoking and often ill health (26). Consequently, the effect of obesity in smokers may be underestimated.

The strength of this study is the use of the more contemporary long-term FOS, where the prevalence of obesity and type-2 diabetes more likely reflects current obesity trends compared with the previous study (9). In FOS, body weight, cofactors, the outcome of diabetes, and mortality were also measured regularly. Most variables were analyzed as time-dependent variables to consider any variations or fluctuations of the value of the variable during the study follow-up. Moreover, a comprehensive number of potential confounders were also adjusted in the analysis, including current smoking status, the number of cigarettes smoked per day, and physical activity.

The main limitation of this study is related to the nature of FOS, for which the interval between examinations was relatively long, between 3 and 8 years. This is in contrast to the FHS, in which the interval between examinations was approximately 2 years. Body weight status and other covariates were not available during these intervals, and this study relied on the assumption that the value was constant during examination intervals. In addition, although we have adjusted for most confounders, some, such as diet, are still not included in the analysis. Moreover, the population of this study is relatively homogeneous and Caucasian. Therefore, this study may not be generalizable to other ethnic populations. Another limitation is that BMI was the only measure of obesity. Prior studies (27, 28) have demonstrated that waist circumference can be a better predictor of metabolic outcome, such as diabetes, which may imply that waist-circumference-years might be a better construct than obese-years.

## Conclusions

Obese-years was confirmed as a better construct for estimating the risk of type-2 diabetes compared with the previous models based solely on either the duration or severity of obesity. The findings also support the use of the obese-years construct for future studies analyzing the total burden of obesity. The burden of obesity may be underestimated if the degree and duration of obesity are not taken into account.

## Paper context

Our previous study demonstrated that an obese-years construct is a better predictor of risk of diabetes than the severity of body weight alone. However, these risk estimates were derived from a relatively old population cohort study of the original FHS that initiated in 1948. Here we validate in a more contemporary cohort study of the FOS. We reconfirmed that the obese-years construct is strongly associated with increased risk of type-2 diabetes. We suggested that obese-years should be considered in future estimations of the burden of disease associated with obesity.
